# Integrated Analysis of Transcriptome and Metabolome Reveals Differential Responses to *Alternaria brassicicola* Infection in Cabbage (*Brassica oleracea* var. *capitata*)

**DOI:** 10.3390/genes15050545

**Published:** 2024-04-25

**Authors:** Jinzhou Lei, Wei Zhang, Fangwei Yu, Meng Ni, Zhigang Liu, Cheng Wang, Jianbin Li, Jianghua Song, Shenyun Wang

**Affiliations:** 1 Anhui Provincial Key Laboratory of Horticultural Crop Quality Biology, College of Horticulture, Anhui Agricultural University, Hefei 230036, China; jinzhoulei2024@163.com (J.L.); 15005608746@163.com (Z.L.); 2 Jiangsu Key Laboratory for Horticultural Crop Genetic Improvement, Institute of Vegetable Crops, Jiangsu Academy of Agricultural Sciences, Nanjing 210014, China; zhangwei@jaas.ac.cn (W.Z.); yfw@jaas.ac.cn (F.Y.); nimeng0715@163.com (M.N.); jbli@jaas.ac.cn (J.L.); 3 Key Laboratory for Quality Control of Characteristic Fruits and Vegetables of Hubei Province, College of Life Science and Technology, Hubei Engineering University, Xiaogan 432000, China; hadesc@hbeu.edu.cn

**Keywords:** cabbage, *Alternaria brassicicola*, reactive oxygen species, jasmonic acid signaling, indolic glucosinolate biosynthesis, transcriptome, metabolome

## Abstract

Black spot, caused by *Alternaria brassicicola* (*Ab*), poses a serious threat to crucifer production, and knowledge of how plants respond to *Ab* infection is essential for black spot management. In the current study, combined transcriptomic and metabolic analysis was employed to investigate the response to *Ab* infection in two cabbage (*Brassica oleracea* var. *capitata*) genotypes, Bo257 (resistant to *Ab*) and Bo190 (susceptible to *Ab*). A total of 1100 and 7490 differentially expressed genes were identified in Bo257 (R_mock vs. R_*Ab*) and Bo190 (S_mock vs. S_*Ab*), respectively. Kyoto Encyclopedia of Genes and Genomes (KEGG) pathway analysis revealed that “metabolic pathways”, “biosynthesis of secondary metabolites”, and “glucosinolate biosynthesis” were the top three enriched KEGG pathways in Bo257, while “metabolic pathways”, “biosynthesis of secondary metabolites”, and “carbon metabolism” were the top three enriched KEGG pathways in Bo190. Further analysis showed that genes involved in extracellular reactive oxygen species (ROS) production, jasmonic acid signaling pathway, and indolic glucosinolate biosynthesis pathway were differentially expressed in response to *Ab* infection. Notably, when infected with *Ab*, genes involved in extracellular ROS production were largely unchanged in Bo257, whereas most of these genes were upregulated in Bo190. Metabolic profiling revealed 24 and 56 differentially accumulated metabolites in Bo257 and Bo190, respectively, with the majority being primary metabolites. Further analysis revealed that dramatic accumulation of succinate was observed in Bo257 and Bo190, which may provide energy for resistance responses against *Ab* infection via the tricarboxylic acid cycle pathway. Collectively, this study provides comprehensive insights into the *Ab*–cabbage interactions and helps uncover targets for breeding *Ab*-resistant varieties in cabbage.

## 1. Introduction

*Alternaria* is a large genus mainly consisting of saprophytic fungi; however, this genus also contains some economically important phytopathogens that cause diseases in a wide range of crops like vegetables, fruits, and cereals [1]. Aside from causing yield losses, *Alternaria* species are known to produce secondary metabolites detrimental for plant growth and human health [2,3]. Among the *Alternaria* species attacking cruciferous crops, *Alternaria brassicae*, *Ab*, *Alternaria alternata*, and *Alternaria raphani* reportedly cause substantial damage [4]. In recent years, there has been an increasing incidence of *Alternaria* black spot (ABS) in the crucifer-growing areas of China, and the development of resistant cultivars is a cost-effective way to control ABS. Unfortunately, the resistant sources imparting complete resistance to ABS are largely unavailable. Only a limited number of resistant sources have been identified in other crucifer species, such as *Camelina sativa*, *Capsella bursa-pastoris*, and *Arabidopsis thaliana* [5,6].

The knowledge of how plants respond to pathogen invasion contributes to the development of strategies for disease management. Rapid production of ROS represents one of the earliest responses upon successful perception of pathogens by plants [7]. The enzymes responsible for extracellular ROS production include cell wall peroxidases, polyamine oxidases, and plasma-membrane-localized NADPH oxidases (respiratory burst oxidase homologs, RBOHs) [8]. The roles of RBOHs and cell wall peroxidases in mediating ABS susceptibility have been documented in *A. thaliana* [6,9]. Disruption of *RBOHD*, *RBOHE*, or *RBOHF* results in reduced ROS accumulation and cell death, thus conferring various degrees of resistance to *A*. *brassicae* [6]. Similarly, knockdown of *PEROXIDASE 33/34* (*PRX33*/*PRX34*) shows reduced colonization of *Ab* [9]. Hormone biosynthesis and signaling pathways are also implicated in plant responses to *Alternaria* infection. *A*. *thaliana* mutants defective in different aspects of the auxin pathway are generally more susceptible to *Ab* as compared with wild-type plants [10]. Disruption of *CORONATINE INSENSITIVE 1* (*COI1*), a gene encoding a critical component of jasmonic acid (JA) signaling, compromises resistance to *Ab* [11,12]. In addition, abscisic acid, ethylene, and salicylic acid signals have also been implicated in plant responses to ABS [13,14]. Plants also produce multiple secondary metabolites to counteract pathogen invasion. For example, the tryptophan-derived defense-related metabolites camalexin and indolic glucosinolates are involved in resistance to ABS [15,16].

To date, our understanding of plant responses to ABS mainly comes from the *Arabidopsis–Alternaria* pathosystem, presumably due to the availability of genetic and genomic resources in combination with well-developed molecular tools in *A*. *thaliana*. Therefore, it is necessary to investigate how other plant species respond to *Alternaria* infection. While genetic manipulation remains a time-consuming and challenging task in many horticultural plants, integrated transcriptome and metabolome profiling may provide another avenue. Recently, integrated analysis of transcriptome and metabolome has been widely used to identify the gene candidates and metabolites playing vital roles in biotic and abiotic stresses and quality-related traits [17,18,19]. In the current study, *Ab*-resistant Bo257 and *Ab*-susceptible Bo190 were selected from 134 cabbage genotypes. The responses of Bo257 and Bo190 to *Ab* infection were then investigated. The differentially expressed genes (DEGs) and differentially accumulated metabolites (DAMs) between the mock and *Ab*-inoculated plants were identified using transcriptomics and widely targeted metabolomics. This study provides comprehensive insights into the interaction between *Ab* and cabbage, aiding in the discovery of targets for breeding *Ab*-resistant varieties. 

## 2. Materials and Methods

### 2.1. Plant Materials, Ab Inoculation, and Sample Collection

Two cabbage genotypes, Bo257 (resistant to *Ab*) and Bo190 (susceptible to *Ab*), were utilized in this study and were provided by the Institute of Vegetable Crops, Jiangsu Academy of Agricultural Sciences. HB1 was a field isolate of *Ab* and was kept at 4 °C before use. The two genotypes (R genotype and S genotype) were grown in a growth chamber at 25 °C under a long-day photoperiod (16 h light/8 h dark). For inoculum preparation, HB1 was cultured on potato dextrose agar (PDA) at 25 °C for 9 days. Subsequently, spores were scraped from sterile distilled-water-flooded PDA plates with a sterile spreader. The resulting suspension was adjusted to 1 × 10^5^ spores mL^−1^ before being uniformly sprayed onto the third true leaf. Plants sprayed with sterile distilled water served as mock controls. The inoculated seedlings were maintained at 25 °C under a relative humidity of approximately 100% for 24 h before being sampled for transcriptomic and metabolomic analysis. The Ab-inoculated samples from Bo257 and Bo190, were labeled as R_*Ab* (R_*Ab*_1, R_*Ab*_2, and R_*Ab*_3) and S_*Ab* (S_*Ab*_1, S_*Ab*_2, and S_*Ab*_3), respectively, while the mock-inoculated samples from Bo257 and Bo190 were labeled as R_mock (R_mock_1, R_mock_2, and R_mock_3) and S_mock (S_mock_1, S_mock_2, and S_mock_3), respectively. All the 12 samples were immediately frozen in liquid nitrogen and sent to Gene Denovo Biotechnology Co., Ltd. (Guangzhou, China), for transcriptomic and metabolomic analysis.

### 2.2. Scanning Electron Microscopy of Cabbage Leaves

Freshly harvested leaves from the two genotypes were carefully washed with distilled water to remove surface dust. The leaves were then cut into pieces measuring approximately 1 cm^2^. Subsequently, fixation was carried out using 2.5% glutaraldehyde for a duration of 2 h. The samples were freeze dried, sputter coated with gold, and finally imaged using Zeiss EVO LS10 scanning electron microscopy (Carl Zeiss, Germany). 

### 2.3. RNA Isolation, Library Construction, and Sequencing

Total RNA was extracted from the cabbage leaves using the Trizol reagent (Invitrogen, USA). Following the RNA quantity and quality assessment, the mRNA was enriched with oligo(dT) beads, and the captured mRNA was fragmented and reversely transcribed into cDNA. The resulting 12 cDNA libraries were sequenced on the Illumina NovaSeq 6000 platform. Three biological replicates were conducted for each experiment. 

### 2.4. Differential Expression Analysis of RNA-Seq Data

Quality control was performed for the raw data by filtering out the adapters, poly-N, and low-quality reads with fastp [20]. The rRNA reads were further removed by mapping with Bowtie2 [21], and the remaining clean reads were then mapped to the cabbage (OX-heart) reference genome using HISAT2 with default parameters [22,23]. The expression levels of the mRNA transcripts were quantified as FPKM (fragments per kilobase of transcript per million mapped reads) using the RSEM v1.3.3 [24]. The DEGs between the treatments (mock and *Ab* inoculation) were identified according to the following criteria: |log_2_(fold change)| > 1 and false discovery rate (FDR) < 0.05 by the DEseq2 package [25]. Subsequently, Gene Ontology (GO) enrichment analysis and KEGG pathway enrichment analysis for DEGs were performed by the clusterProfiler R package [26], in which the top 20 most represented categories were presented. Heatmaps were generated using TBtools v1.098661 [27].

### 2.5. Widely Targeted Metabolomic Analysis

The leaf samples were finely ground in liquid nitrogen, and then approximately 100 mg of the powder was subjected to extraction for 12 h at 4 °C with 1.0 mL of 70% methanol solution. After centrifugation, the supernatant was collected and filtered as previously described [28]. The metabolites of the 12 samples were analyzed using a liquid chromatography–electrospray ionization tandem mass spectrometry (LC-ESI-MS/MS) system. The acquired metabolomics data were analyzed according to previously reported methods [29]. DAMs between treatments (mock and *Ab* inoculation) were screened with a *t*-test *p* < 0.05 and variable importance in projection (VIP) ≥1.

## 3. Results

### 3.1. Distinct Disease Symptoms in Leaves of Bo257 and Bo190 after Ab Infection

**Figure 1:**
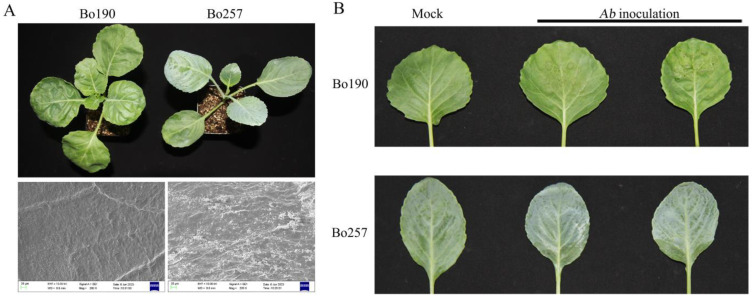
Observation of leaf surface and disease symptom development in Bo257 and Bo190. (**A**), scanning electron microscopy results of leaf surface of Bo257 and Bo190. (**B**), disease development in Bo257 and Bo190. Pictures were taken at 24 hpi.

Under the same growth conditions, the leaf of Bo190 (S genotype) showed a shiny green color, while that of Bo257 (R genotype) exhibited a pallid green color (Figure 1A). Examination of the leaf surface by scanning electron microscopy showed that more epicuticular wax was observed on the leaves of Bo257 as compared with those of Bo190 (Figure 1A). To verify the differential responses to *Ab* infection in the two cabbage lines, leaves of Bo257 and Bo190 seedlings were artificially inoculated with *Ab*. At 24 h post inoculation (hpi), dark spots were clearly visible on the leaf surface of Bo190, while only a few dark spots were observed on that of Bo257 (Figure 1B), suggesting that the development of black spot symptoms is somehow delayed in Bo257.

### 3.2. Identification of DEGs after Ab Infection

In order to explore the gene regulatory networks associated with resistance to black spot, a total of 12 RNA samples, collected from three biological replicates of mock and *Ab*-inoculated leaves of Bo257 and Bo190 at 24 hpi, were subjected to RNA-Seq analysis. Approximately 532.7 million raw reads were generated, yielding an average of 44.4 million reads per sample (Table S1). After quality filtering, approximately 530.5 million reads were obtained. After the removal of rRNA reads, the remaining reads (approximately 528.3 million reads) were aligned to the OX-heart genome [22]. The majority of the reads (approximately 474.2 million) could be mapped uniquely to one location within the reference genome, while a small portion of the reads were either mapped multiple times (approximately 16.3 million reads) or unmapped (approximately 37.8 million reads) (Table S1). The majority of the genes of the biological replicates were clustered together, as revealed by principal components analysis and Pearson’s correlation analysis (Figures S1 and S2).

**Figure 2:**
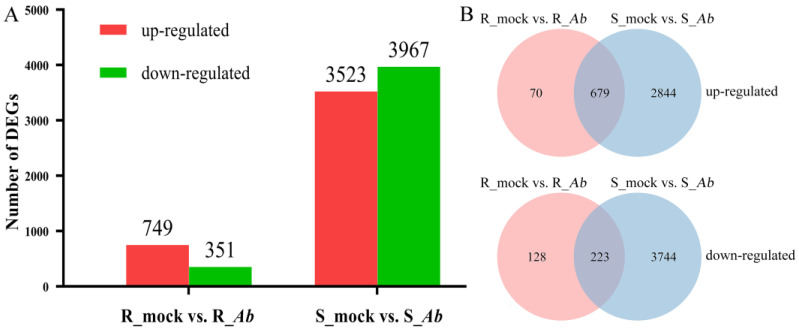
Identification of DEGs in Bo257 (R_mock vs. R_*Ab*) and Bo190 (S_mock vs. S_*Ab*) after *Ab* infection. (**A**) Number of DEGs. (**B**) Common and unique DEGs.

A total of 1100 and 7490 DEGs were identified in Bo257 (R_mock vs. R_*Ab*) and Bo190 (S_mock vs. S_*Ab*), respectively (Figure 2A). After *Ab* infection, 749 DEGs were upregulated and 351 DEGs were downregulated in the R-genotype Bo257 (Figure 2A). Different from Bo257, 3523 DEGs were upregulated and 3967 DEGs were downregulated in the S-genotype Bo190 (Figure 2A). Of all the upregulated DEGs identified, 679 DEGs were shared by Bo257 and Bo190, while 70 and 2844 DEGs were uniquely identified in Bo257 and Bo190, respectively (Figure 2B). As for the downregulated DEGs identified, 223 DEGs were shared by Bo257 and Bo190, while 128 and 3744 DEGs were uniquely identified in Bo257 and Bo190, respectively (Figure 2B).

### 3.3. Functional Annotation of DEGs

**Figure 3:**
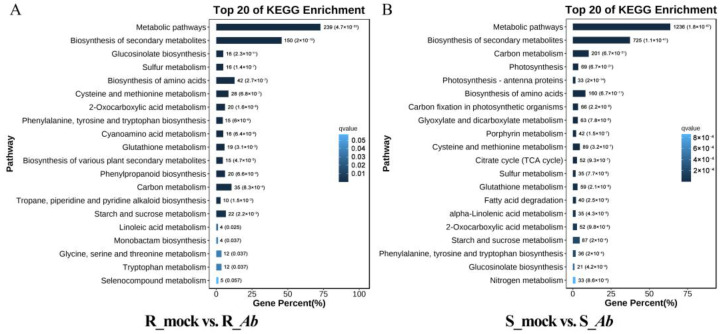
KEGG pathway enrichment analysis of DEGs after *Ab* infection. (**A**) Top 20 enriched KEGG pathways in Bo257 (R_mock vs. R_*Ab*). (**B**) Top 20 enriched KEGG pathways in Bo190 (S_mock vs. S_*Ab*).

GO term enrichment analysis was performed to classify their gene functions according to three main GO terms: biological process (BP), cellular component (CC), and molecular function (MF). For the BP ontology, the main enriched terms were “cellular process”, “metabolic process”, and “response to stimulus” (Figure S3). To better understand the biological significance of the gene functions of the identified DEGs, these DEGs were mapped to reference canonical pathways in the KEGG database. Among the top 20 enriched KEGG pathways, 11 were shared by Bo257 and Bo190, including “metabolic pathways”, “biosynthesis of secondary metabolites”, “glucosinolate biosynthesis”, “sulfur metabolism”, “biosynthesis of amino acids”, “cysteine and methionine metabolism”, “2-oxocarboxylic acid metabolism”, “phenylalanine, tyrosine and tryptophan biosynthesis”, “glutathione metabolism”, “carbon metabolism”, and “starch and sucrose metabolism” (Figure 3A,B). Nine pathways, including “cyanoamino acid metabolism”, “biosynthesis of various plant secondary metabolites”, “phenylpropanoid biosynthesis”, “tropane, piperidine and pyridine alkaloid biosynthesis”, “linoleic acid metabolism”, “monbactam biosynthesis”, “glycine, serine and threonine metabolism”, “tryptophan metabolism”, and selenocompound metabolism” were enriched in Bo257 (Figure 3A). By contrast, pathways including “photosynthesis”, “photosynthesis-antenna proteins”, “carbon fixation in photosynthetic organisms”, “glyoxylate and dicarboxylate metabolism”, “porphyrin metabolism”, “tricarboxylic acid (TCA) cycle”, “fatty acid degradation”, “α-Linolenic acid metabolism”, and “nitrogen metabolism” were enriched in Bo190 (Figure 3B).

### 3.4. Differential Expression of Genes Involved in Extracellular ROS Production

**Figure 4:**
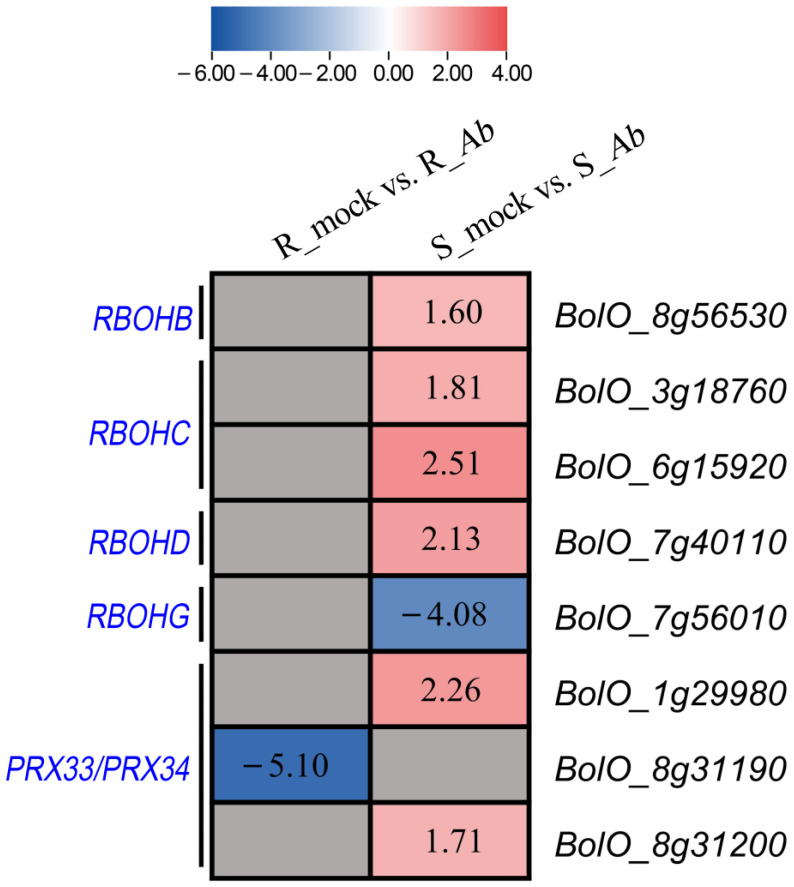
Differential expression of genes involved in extracellular ROS production in Bo257 (R_mock vs. R_*Ab*) and Bo190 (S_mock vs. S_*Ab*) after *Ab* infection. Values in the heatmap are shown as log_2_ fold changes. Grey box without value indicates no differential expression between the mock and *Ab* inoculation.

Rapid production of ROS represents one of the earliest responses upon the successful perception of pathogens by plants [7]; therefore, we first investigated the transcript abundance of genes responsible for extracellular ROS production. A total of 23 homologues were identified in OX-heart genome, with 20 genes encoding cell wall peroxidases and 3 encoding plasma-membrane-localized NADPH oxidases (Table S2). Interestingly, one cabbage homologue (*BolO_8g31190*) of *PRX33*/*PRX34* was downregulated in Bo257 (Figure 4). By contrast, six cabbage homologues, *BolO_8g56530* (homologue of *RBOHB*), *BolO_3g18760*, and *BolO_6g15920* (homologue of *RBOHC*), *BolO_7g40110* (homologue of *RBOHD*), *BolO_1g29980*, and *BolO_8g31200* (homologue of *PRX33*/*PRX34*) were upregulated, while one gene *BolO_7g56010* (homologue of *RBOHG*) was downregulated in Bo190 (Figure 4). 

### 3.5. Differential Expression of Genes Involved in JA Signaling Pathway

**Figure 5:**
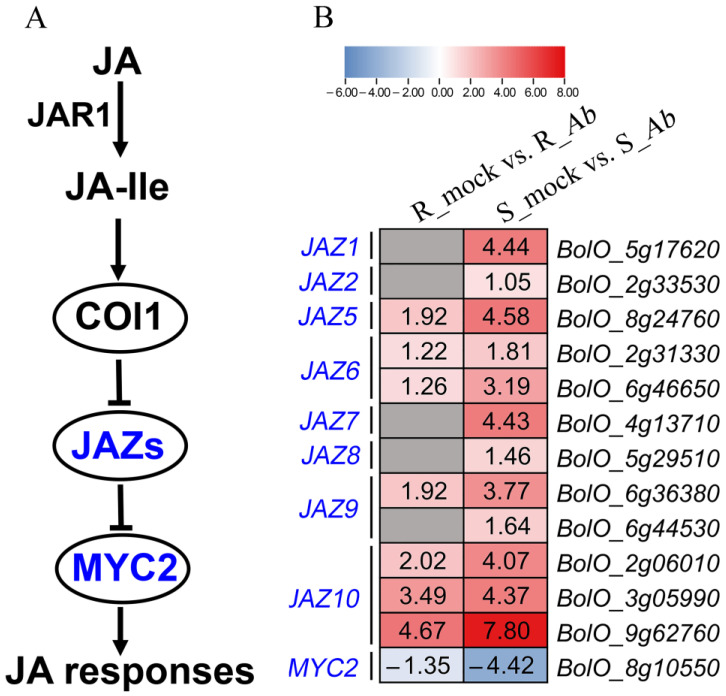
Differential expression of genes involved in JA signaling pathway in Bo257 (R_mock vs. R_*Ab*) and Bo190 (S_mock vs. S_*Ab*) after *Ab* infection. (**A**) The JA signaling pathway. JA-Ile: jasmonoyl-isoleucine; JAR1: JASMONATE RESISTANT 1. (**B**) Expression profiles of genes involved in the JA signaling pathway. Values in the heatmap are shown as log_2_ fold changes. Grey box without value indicates no differential expression between the mock and *Ab* inoculation.

Depending on the distinct strategies adopted by plant pathogenic fungi, they are divided into biotrophs, hemibiotrophs, and necrotrophs. As a necrotrophic fungus, *Ab* infects host tissue and extracts nutrients from dead host cells. JA signaling represents one of the most studied pathways in terms of plant–necrotrophic fungus interaction and thus was reconstructed based on previous studies [30,31,32] (Figure 5A). Therefore, a total of 34 genes responsible for JA signaling pathway were subjected to an examination of transcript abundance in the current study (Table S2). As shown in Figure 5B, thirteen genes were differentially expressed in Bo190, albeit to varying degrees, while only eight DEGs were identified in Bo257. It is well known that JASMONATE ZIM-DOMAIN (JAZ) proteins are key regulators in the JA signaling pathway and function as transcription repressors of JA-responsive genes [33,34]. Interestingly, 12 genes encoding JAZ protein-encoding were upregulated, including *JAZ1* (*BolO_5g17620*), *JAZ2* (*BolO_2g33530*), *JAZ5* (*BolO_8g24760*), *JAZ6* (*BolO_2g31330* and *BolO_6g46650*), *JAZ7* (*BolO_4g13710*), *JAZ8* (*BolO_5g29510*), *JAZ9* (*BolO_6g36380* and *BolO_6g44530*), and *JAZ10* (*BolO_2g06010*, *BolO_3g05990*, and *BolO_9g62760*), while the expression of *MYC2* (*BolO_8g10550*) was decreased in Bo190 (Figure 5B). The *Ab* inoculation also resulted in an increased expression of *JAZ5*, *JAZ6*, *JAZ9*, and *JAZ10* and a decreased expression of *MYC2* in Bo257 but to a lesser degree than those in Bo190 (Figure 5B).

### 3.6. Differential Expression of Genes Involved in Indolic Glucosinolate Biosynthesis Pathway

**Figure 6:**
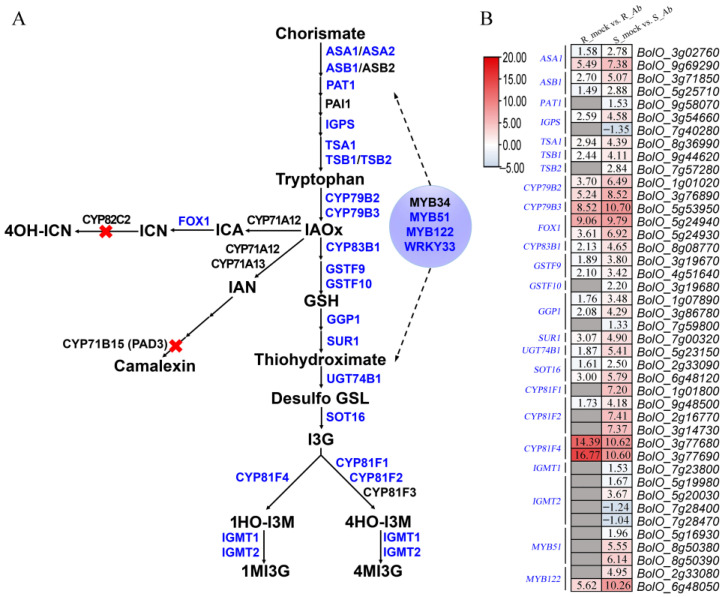
Differential expression of genes involved in indolic glucosinolate biosynthesis pathway in Bo257 (R_mock vs. R_*Ab*) and Bo190 (S_mock vs. S_*Ab*) after *Ab* infection. (**A**) The indolic glucosinolate biosynthesis pathway. IAOx: indole-3-acetaldoxime; ICA: indole-3-carboxylic acid; ICN: indole-3-carbonylnitrile; 4OH-ICN: 4-hydroxy-indole-3-carbonylnitrile; IAN: indole-3-acetonitrile; GSH: glutathione; Desulfo GSL: desulfoglucosinolate; I3G: indole-3-ylmethyl glucosinolate; 1HO-I3M: 1-hydroxy-indole-3-ylmethyl glucosinolate; 4HO-I3M: 4-hydroxy-indole-3-ylmethyl glucosinolate; 1MI3G: 1-methoxyindole-3-ylmethyl glucosinolate; 4MI3G: 4-methoxyindole-3-ylmethyl glucosinolate. Red X symbol indicates the loss of 4OH–ICN biosynthetic gene *CYP82C2* and camalexin biosynthetic gene *CYP71B15* (*PAD3*) in cabbage. (**B**) Expression profiles of genes involved in the indolic glucosinolate pathway. Values in the heatmap are shown as log_2_ fold changes. Grey box without value indicates no differential expression between the mock and *Ab* inoculation.

Glucosinolates, especially indolic glucosinolates, play an important role in the resistance to *Ab* infection in *Arabidopsis*, Chinese kale, and broccoli [15,16]; thus, we were interested to investigate the expression profiles of the 72 genes involved in the biosynthesis pathway of indolic glucosinolates in cabbage (Table S2). First, the indolic glucosinolate biosynthesis pathway was reconstructed based on previous studies [35,36,37] (Figure 6A). A total of 42 DEGs were identified in Bo190, containing 39 upregulated DEGs and 3 downregulated DEGs (Figure 6B). By contrast, 25 DEGs were upregulated in Bo257 after *Ab* infection (Figure 6B). Overall, the expression of genes involved in the biosynthesis pathway of indolic glucosinolates in Bo257 was either unchanged or lower than that in Bo190. However, *BolO_3g77680* and *BolO_3g77690*, homologues of *CYP81F4* responsible for the conversion of indole-3-yl-methyl glucosinolate (I3M) to 1-hydroxyindol-3-ylmethyl glucosinolate (1HO-I3M), showed a higher expression in Bo257 (Figure 6B). 

### 3.7. Identification of DAMs after Ab Infection 

**Figure 7:**
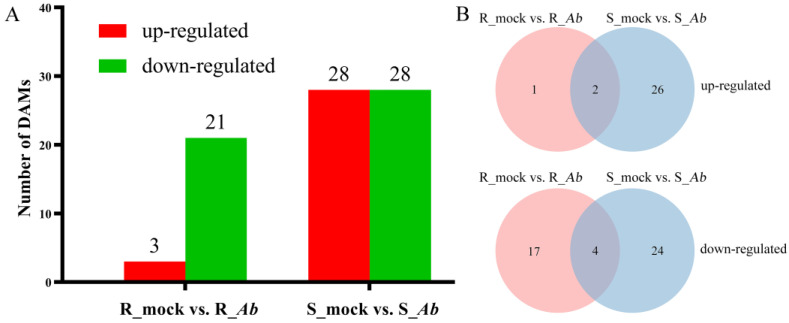
Identification of DAMs in Bo257 (R_mock vs. R_*Ab*) and Bo190 (S_mock vs. S_*Ab*) after *Ab* infection. (**A**) Number of DAMs. (**B**) Common and unique DAMs.

**Figure 8:**
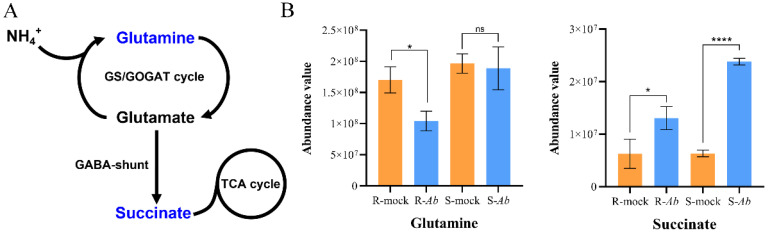
Analysis of the glutamate metabolism in Bo257 (R_mock vs. R_*Ab*) and Bo190 (S_mock vs. S_*Ab*). (**A**) Schematic representation of glutamate metabolism. GS: glutamine synthetase; GOGAT: glutamine 2-oxoglutarate aminotransferase; GABA: γ-aminobutyrate. (**B**) Comparison of the glutamine and succinate abundance value in Bo257 and Bo190 after *Ab* infection. Data are presented as means ± standard deviation (SD) from three independent biological replicates. Statistical analysis was performed using Student’s two-tailed *t*-test by GraphPad Prism 8. Asterisks indicate statistically significant differences between treatments or genotypes. *: *p* < 0.05, ****: *p* < 0.0001, ns: not significant.

In order to explore the metabolic changes in cabbage after *Ab* infection, metabolic profiles of the R-genotype Bo257 and S-genotype Bo190 were analyzed by the LC-ESI-MS/MS method. After quality control, a total of 1112 metabolites were identified from the tested 12 samples. Among them, a total of 24 and 56 DAMs were identified from Bo257 (3 DAMs were upregulated and 21 DAMs were downregulated) and Bo190 (28 DAMs were upregulated and 28 DAMs were downregulated), respectively (Figure 7A). Of all the upregulated DAMs identified, only 2 DAMs were shared by Bo257 and Bo190, while 1 and 26 DAMs were uniquely identified in Bo257 and Bo190, respectively (Figure 7B). As for the downregulated DAMs identified, only 4 DAMs were shared by Bo257 and Bo190, while 17 and 24 DAMs were uniquely identified in Bo257 and Bo190, respectively (Figure 7B). These six DAMs shared by Bo257 and Bo190 were N-methyl-a-aminoisobutyric acid, succinate, methylmalonate, L-asparagine, leucine, and ureidosuccinic acid (Tables S3 and S4). These 74 DAMs were classified into 13 categories, including 36 amino acids and their derivatives, 3 carbohydrates and their derivatives, 9 organic acids and their derivatives, 2 vitamins, 4 nucleotides and their derivates, 4 lipids, 5 organoheterocyclic compounds, 5 alkaloids and their derivatives, 2 phenolic acids, 1 amine, 1 polyamine, 1 phytohormone, and 1 organosulfur compound (Tables S3 and S4). The abundance of DAMs from nine major categories was analyzed (Figure S4). We found that Bo257 and Bo190 contained more primary metabolites (20 and 44) than the secondary metabolites (4 and 12), respectively (Tables S3 and S4). In addition, 50 unique DAMs (26 upregulated and 24 downregulated) in Bo190 were identified. The glutamate-metabolism-related γ-aminobutyrate (GABA) shunt was reconstructed based on previous studies [38,39] (Figure 8A). Compared with R_mock, the accumulation of glutamine was decreased in R_*Ab*, while this metabolite was not significantly changed in S_*Ab* as compared with S_mock. The accumulation of succinate was significantly increased in both R_*Ab* and S_*Ab*, albeit to different degrees (Figure 8B). 

## 4. Discussion

Black spot caused by *Ab* is an economically important disease of cabbage. The knowledge of how other plants besides *Arabidopsis* respond to *Ab* infection will be helpful to aid in the control of ABS. In the current study, *Ab*-resistant Bo257 was selected from 134 cabbage accessions. Compared with the susceptible genotype Bo190, more epicuticular wax was observed on the leaf surface of Bo257 (Figure 1A). Meanwhile, when inoculated with *Ab*, fewer dark spots were visible on the leaf surface of Bo257 (Figure 1B). The cuticular waxes are mainly composed of hydrophobic compounds, including fatty acids and their derivatives such as alkanes, aldehydes, primary alcohols, alky esters, secondary alcohols, and ketones [40]. The roles of cuticular wax in plant defense have been explored in many pathosystems. Epicuticular wax is positively correlated with resistance to *A*. *brassicae* in rapeseed and mustard [41]. However, a different scenario was observed in the interaction between *Brassica napus* and another necrotrophic fungus, *Sclerotinia sclerotiorum*, where the total amount of wax was significantly lower in the resistant cultivar compared with that in the susceptible cultivar [42]. Sorghum leaf wax improves the growth of *Penicillium* but suppresses *A. alternata*, while sheath wax suppresses *Penicillium* but does not affect *A. alternata* [43]. In *Arabidopsis*, the disruption of the *LTPG1* gene, encoding a plasma-membrane-localized lipid transfer protein, leads to alterations in cuticular lipid composition but has no significant impact on total wax and cutin monomer loads [44]. Notably, the mutant shows increased susceptibility to *Ab* than the wild type [44]. 

Previous studies have suggested that multiple mechanisms are involved in plant resistance against *Alternaria* species [16,45]. Therefore, to unveil the possible involvement of other regulatory networks in the interaction between cabbage and *Ab*, the transcriptional landscape was investigated in the *Ab*-resistant genotype Bo257 and the *Ab*-susceptible genotype Bo190 that were exposed to *Ab* infection. Based on *Ab* infection cycle studies on *Brassica oleracea* leaves [46,47] and our results (Figure 1B), inoculated cabbage leaves at 24 hpi were sampled for analysis of their transcriptome and metabolome. Interestingly, when infected with *Ab*, only 1100 DEGs were detected in Bo257 (R_mock vs. R_*Ab*), while 7490 DEGs were identified in Bo190 (S_mock vs. S_*Ab*) (Figure 2). In response to *Ab* invasion, 3844 DEGs were detected in a resistant broccoli, whereas only 1616 DEGs were detected in a susceptible broccoli [16]. In another study, when infected with *Xanthomonas campestris* pv. *campestris*, 624 DEGs were detected at 2 dpi in a resistant cabbage line, while 3040 DEGs were detected at 2 dpi in a susceptible cabbage line [19]. GO term enrichment analysis showed that the main enriched terms were “cellular process”, “metabolic process”, and “response to stimulus” (Figure 3A). Furthermore, analysis of enriched KEGG pathways revealed that “metabolic pathways”, “biosynthesis of secondary metabolites”, and “glucosinolate biosynthesis” were the top three enriched pathways in *Ab*-infected Bo257, while “metabolic pathways”, “biosynthesis of secondary metabolites”, and “carbon metabolism” were the top three enriched pathways in *Ab*-infected Bo190 (Figure 3B). An essential part of plant apoplastic defense is the production of ROS by the action of RBOHs and cell wall peroxidases [48,49,50]. Of all the 23 gene homologues of *RBOHs* and *PRX33*/*PRX34* predicted in cabbage (Table S2), only one homologue (*BolO_8g31190*) of *PRX33*/*PRX34* was downregulated in Bo257 after *Ab* infection (Figure 4). By contrast, six genes, including *RBOHB* (1), *RBOHC* (2), *RBOHD* (1), and *PRX33*/*PRX34* (2), were upregulated with only one homologue of *RBOHG* being downregulated in Bo190 (Figure 4). In *Arabidopsis*, disruption of *RBOHs* or *PRX33*/*PRX34* results in various degrees of resistance to *A. brassicae* or *Ab* infection [6,9], indicating their negative roles in the *Arabidopsis*–*Alternaria* interaction. Therefore, it is reasonable to hypothesize that the accumulation of extracellular ROS is not beneficial for cabbage to combat *Ab* infection. JA signaling is an important part of plant defense against necrotrophic fungus. Surprisingly, JA signaling was somehow inhibited by *Ab* infection in both Bo257 and Bo190 because an increased expression of *JAZ* homologues and a decreased expression of *MYC2* homologues were observed in both genotypes, albeit to varying degrees (Figure 5B). Further study is needed to clarify the involvement of JA signaling in the cabbage response to *Ab* infection. The role of indolic glucosinolates in the resistance to *Ab* infection has been documented in *Arabidopsis*, Chinese kale, and broccoli [15,16]. A total of 25 and 39 genes involved in the biosynthesis pathway of indolic glucosinolates were upregulated in response to *Ab* infection in Bo257 and Bo190, respectively (Figure 6B). Notably, *BolO_3g77680* and *BolO_3g77690*, homologues of *CYP81F4* responsible for the conversion of indole-3-yl-methyl glucosinolate (I3M) to 1-hydroxyindol-3-ylmethyl glucosinolate (1HO-I3M), showed a higher expression in Bo257 (Figure 6B).

When confronted with biotic stresses, the host plants also undergo metabolic remodeling. Metabolic profiling revealed 24 and 56 DAMs in Bo257 and Bo190, respectively (Figure 7A). This was in agreement with the transcriptomic analysis that showed that a smaller number of DEGs were detected in Bo257 than that in Bo190 (Figure 2), suggesting that Bo257 is much more stable both transcriptionally and metabolically than Bo190 after *Ab* infection. It is therefore interesting to further investigate the mechanism that Bo257 employs to maintain homeostasis after *Ab* infection. A total of 20 and 44 differentially accumulated primary metabolites were detected in Bo257 and Bo190, respectively, while 4 and 12 differentially accumulated secondary metabolites were detected in Bo257 and Bo190, respectively (Tables S3 and S4), suggesting the possible role of primary metabolism in plant response to *Ab* infection. It has been proposed that the role of primary metabolism during plant–pathogen interactions is to maintain cellular energy requirements in host plant defense responses [51,52]. The GABA shunt pathway is able to convert GABA into succinate, thereby connecting the glutamine synthetase (GS)/glutamine 2-oxoglutarate aminotransferase (GOGAT) cycle to the TCA cycle (Figure 8A). The GABA shunt increases succinate formation via three key enzymes: glutamate decarboxylase, GABA transaminase, and succinic semialdehyde dehydrogenase [53]. In the present study, the accumulation of succinate was significantly upregulated in Bo257 and Bo190 after *Ab* infection, coinciding with a reduced level of glutamine (Figure 8B). The glutamate-metabolism-related TCA cycle pathway plays a critical anabolic role in plant defense against pathogens because the resistance responses are known as highly energy-demanding processes in plants [38,54,55]; therefore, the replenishment of the TCA cycle with succinate presumably provides energy for defense against *Ab* infection in cabbage.

## 5. Conclusions

In this study, we found that Bo257 accumulated more epicuticular wax than Bo190, which may contribute partially to *Ab* resistance. In addition, our work revealed that genes responsible for extracellular ROS production were not actively expressed, while most genes involved in the biosynthesis pathway of indolic glucosinolates were upregulated during *Ab* infection. Furthermore, Bo257 was able to minimize the damage caused by *Ab* infection by maintaining metabolic homeostasis. 

## Data Availability

The raw sequencing data have been deposited in the Genome Sequence Archive in National Genomics Data Center (https://ngdc.cncb.ac.cn/gsa, accessed on 21 December 2023) under accession number CRA014095.

## Supplementary Materials

The following supporting information can be downloaded at: https://www.mdpi.com/article/10.3390/genes15050545/s1, Figure S1. Principal component analysis of the 12 RNA-Seq samples; Figure S2. Pearson’s correlation analysis of the 12 RNA-Seq samples; Figure S3. GO enrichment analysis of DEGs in Bo257 (R_mock vs. R_*Ab*) and Bo190 (S_mock vs. S_*Ab*) after *Ab* infection; Figure S4. Comparison of the metabolite abundance value in Bo257 (R_mock vs. R_*Ab*) and Bo190 (S_mock vs. S_*Ab*) after *Ab* infection; Table S1. Summary of RNA-Seq data for 12 samples; Table S2. The expression of genes involved in extracellular ROS production, JA signaling pathway, and indolic glucosinolate biosynthesis pathway in Bo257 (R_mock vs. R_*Ab*) and Bo190 (S_mock vs. S_*Ab*) after *Ab* infection; Table S3. DAMs identified in Bo257 (R_mock vs. R_*Ab*) after *Ab* infection; Table S4. DAMs identified in Bo190 (S_mock vs. S_*Ab*) after *Ab* infection.
