# Supplementary material for: Integrated Analysis of Transcriptome and Metabolome Reveals Differential Responses to Alternaria brassicicola Infection in Cabbage (Brassica oleracea var. capitata)
Source: Genes (Basel). 2024 Apr 25;15(5):545. doi: 10.3390/genes15050545 (PMC11121261; doi:10.3390/genes15050545)
Supplement: Supplementary file 1 [file genes-15-00545-s001.zip › Supplementary figures S1-S4.pdf]

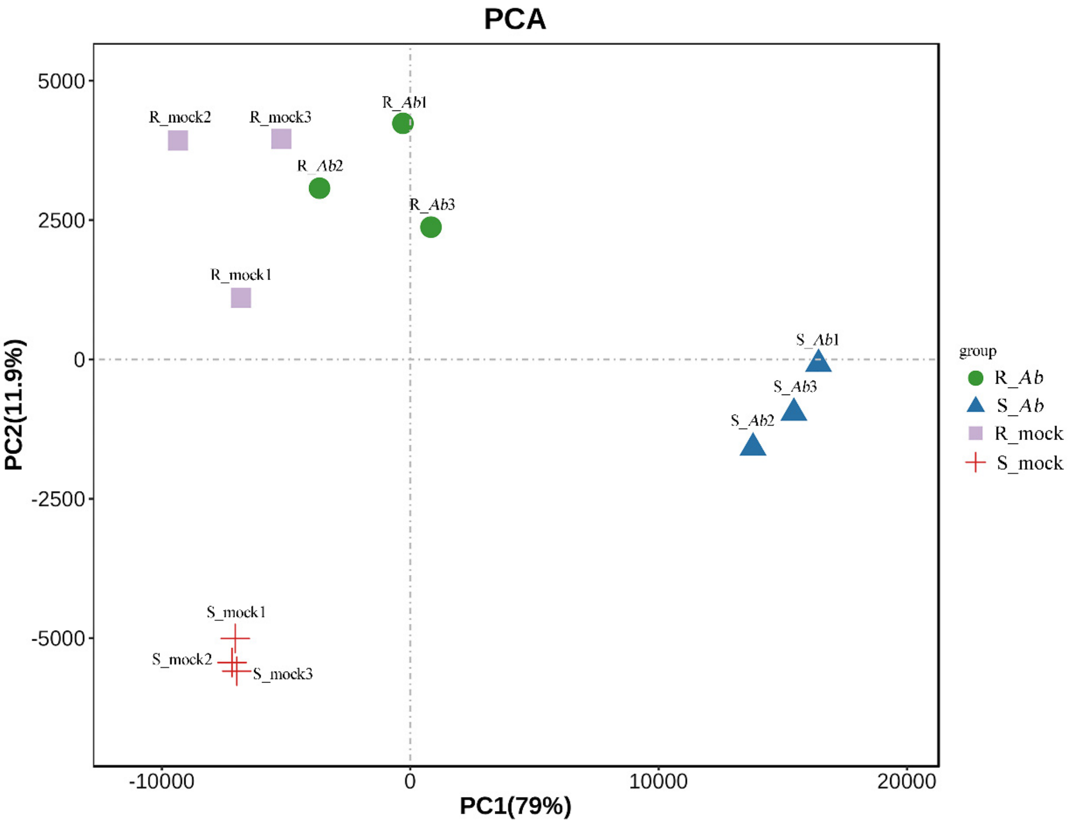

Figure S1. Principal component analysis of the12 RNA-Seq samples.

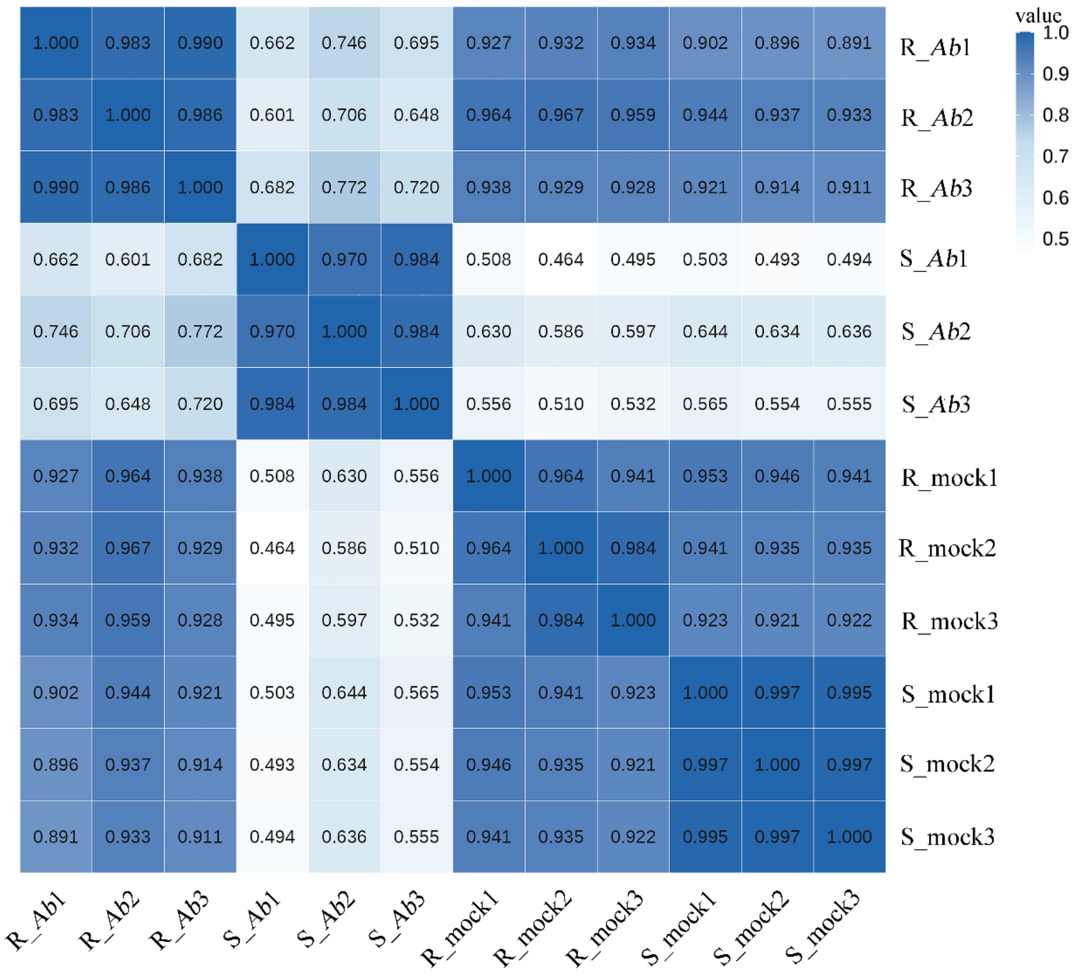

Figure S2. Pearson correlation analysis of the 12 RNA-Seq samples.

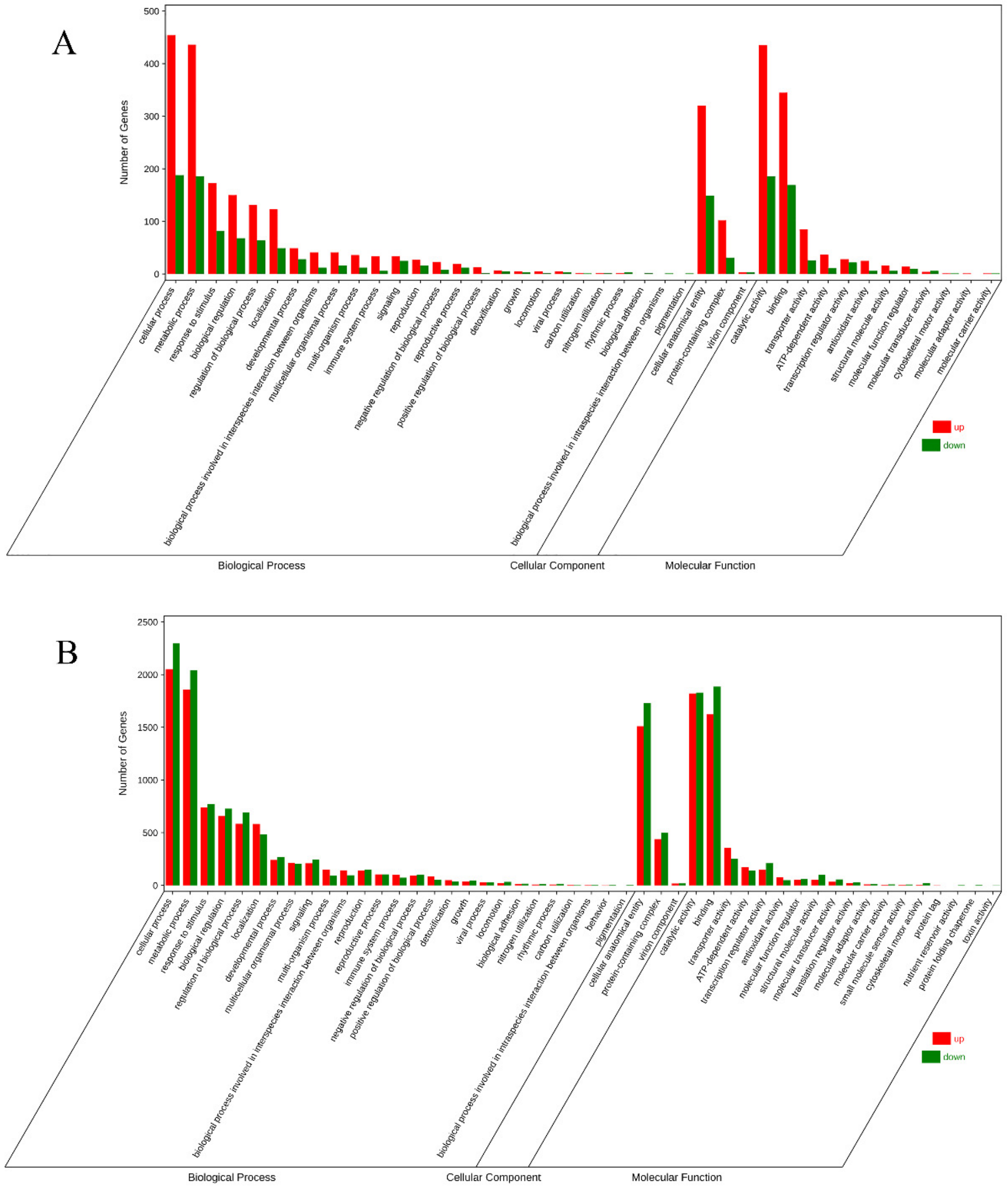

Figure S3. GO enrichment analysis of DEGs in Bo257 (R_mock vs. R_*Ab*) and Bo190 (S_mock vs. S_*Ab*) after *Ab* infection. A, GO enrichment analysis of DEGs in Bo257 (R_mock vs. R_*Ab*). B, GO enrichment analysis of DEGs in Bo190 (S_mock vs. S_*Ab*). The y-axis represents the total number of DEGs annotated to each GO process. The x-axis represents three ontologies, including biological processes, molecular functions, and cellular components. The red and green sections represent up-regulated and down-regulated DEGs, respectively.

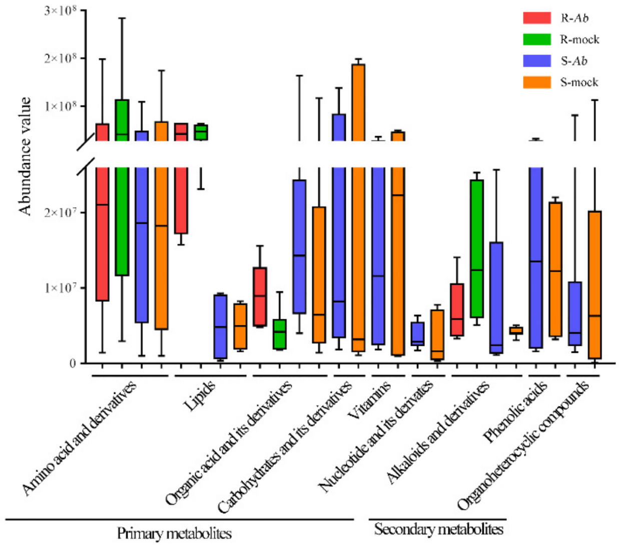

Figure S4. Comparison of the metabolite abundance value in Bo257 (R_mock vs. R_*Ab*) and Bo190 (S_mock vs. S_*Ab*) after *Ab* infection.
